# Clockwork precision: egg-laying-induced rise of body temperature is seasonally programmed in a wild bird

**DOI:** 10.3389/fphys.2024.1490877

**Published:** 2024-11-13

**Authors:** Magella Guillemette, Yannick Seyer, Anouck Viain

**Affiliations:** ^1^ Département de Biologie, Chimie et Géographie, Université du Québec à Rimouski, Rimouski, QC, Canada; ^2^ Département de Biologie, Chimie et Géographie, Université du Québec à Rimouski, Rimouski, QC, Canada; ^3^ Ligue Pour la Protection des Oiseaux (LPO BFC), Délégation Territoriale Franche-Comté, Besançon, France

**Keywords:** body temperature, laying phenology, reproduction, photoperiod, seasonal variation, ambient temperature, common eider, *Somateria mollissima*

## Abstract

There is long time interest about the phenology of plants and animals living in seasonal environments as research in that field would help to understand the coping mechanisms leading to a higher fitness. For instance, it has been shown several decades ago that birds prepare themselves 2–4 months before the actual start of the breeding season by slowly growing reproductive organs. In parallel, the resting metabolic rate increase during reproduction in various vertebrates including mammals, reptiles, and birds. Recently, it has been reported that body temperature of a marine bird species was reaching an annual peak during egg-laying, raising the question about the seasonal dynamic of this important physiological feature. Using data loggers implanted in the abdominal cavity of female Common Eiders (*Somateria mollissima mollissima*) for a full year, we show here that daily body temperature (*T*_b.daily_) is slowly increasing first and then accelerating at the approach of the laying period. Because the rise of *T*_b.daily_ is tightly associated with egg-laying in this species, we also analysed the influence of ambient temperature (water and air) and photoperiod on this seasonal dynamic. Based on the various mechanisms at work and a parsimonious interpretation of the data, we conclude that photoperiod is the main cue driving the seasonal breeding program of eiders. Although the laying dates of the instrumented females were highly clustered over a period of 4 years, we speculated that the remaining variation observed was the result of eco-physiological challenges occurring over the years.

## Introduction

Seasonality has shaped the natural history of various life forms as it has a profound effect on the availability of resources and ambient temperature. In parallel, most living organisms developed a sophisticated array of adaptations in order to anticipate periods of high and low resource levels together with the actual environment (Bradshaw and Holzapfel, 2007; Paul et al., 2008). For this reason, the occurrence of various biological phases of birds, including migration, moulting and reproduction, have been investigated several times from a phenological point of view in the past (Ramenofsky et al., 2012; Dixit et al., 2020). Reproduction is the most frequent target in that regard, probably because it contributes directly to fitness.

Nine decades ago, it was shown that reproductive organs of male and female European Starlings *Sturnus vulgaris* start to increase slowly as early as mid-January (Bissonnette, 1931; Bissonnette and Zujko, 1936). These findings were confirmed by various studies thereafter showing that reproductive organs of birds start to grow a long time before the actual reproductive season in the northern hemisphere (Farner, 1964; King et al., 1966; Tewary and Ravikumar, 1989; Silverin et al., 1993; Jain and Kumar, 1995; Huysentruyt et al., 2013). In parallel, resting metabolism increases during reproduction in various vertebrates, including placental and marsupial mammals, reptiles, and birds (Farmer, 2000; Koteja, 2000). Recently, Guillemette and Pelletier (2022) showed that the period of egg-laying of Common Eider (*Somateria mollissima*), a large sea duck, was associated with an annual peak of body temperature (*T*_b_). Although the authors hypothesised that a high *T*_b_ during egg-laying would be adaptive and improve the fertilisation process, it raises the question if such a level of thermogenesis would be activated in advance of the breeding season as observed for reproductive organs?

Available daylight (photoperiod) is the most widespread cue used by wild birds to prepare themselves prior to the breeding season (Farner, 1964; Dawson, 2007; Ramenofsky, 2012; Dixit et al., 2020). This is because photoperiod is a cue of a high predictive value that seasonally varies in a similar fashion at a given latitude. Although, photoperiod seems to be the main driver of seasonality of birds (together with an endogenous circannual clock, see Gwinner and Scheuerlein, 1998; Helm et al., 2017), other local supplementary cues like ambient temperature (Silverin et al., 2008), food abundance (Poulin et al., 1992; Hahn, 1998), and precipitation (Perfito et al., 2007) can modulate further the exact time at which breeding and egg-laying period occurs. For instance, ambient (water or air) temperature variation does not always correlate tightly with photoperiod and given the pervasive effect of temperature on animal and plant life, a photoperiod-temperature interaction is expected once on the breeding grounds (Wingfield et al., 1997; 2003; Silverin et al., 2008; Ramenofsky, 2012).

Using data loggers implanted in the abdominal cavity of female Common Eiders (*Somateria mollissima mollissima*) for a full year, we present evidence that *T*_b_ is increasing in a seasonal fashion to reach a peak during the egg-laying period. Because the mechanisms triggering the increase of *T*_b_ are unknown, we aimed to analyse in a second step the consistency of egg-laying phenology over the years and investigate if sea surface temperature (SST), air temperature (*T*_air_) and photoperiod are related to the seasonal *T*_b_ variation observed.

## Materials and methods

### Study site

This study was performed on Christiansø Island (55°19′N, 15°12′E), located in the southern Baltic Sea, 18 km from the Danish island of Bornholm. One of the reasons we chose this colony is that habituation of the birds to human presence has developed over the years, facilitating monitoring at the colony, handling and re-capturing the experimental birds (see Guillemette and Pelletier, 2022). Our study required daily visits to the colony to monitor clutch size and laying phenology, and for the implantation of data loggers into incubating females.

### Capture and data logger implantation

We obtained a license from Dyreforsøgtilsynet (Royal Veterinarian Corporation) in Denmark, where the data loggers were deployed. All birds were cared for in accordance with the principles and guidelines of the Canadian Council on Animal Care (CPA #16-03-07-01). Our study was conducted on females only as data presented here are based on archival data-loggers which require to catch back the same individual 1 year later and males, which are not faithful to the same nest, are much harder to capture. Although males have a different reproductive biology than females, we believe our results also apply to males as they arrive in synchrony with females at the colony (Franzmann, 1980). Between 2003 and 2006, we surgically implanted data loggers (36 mm long × 28 mm wide × 11 mm thick and weighed 21 g) in the abdominal cavity of 45 experimental female Common Eiders. These loggers were engineered and constructed by Tony Woakes from Birmingham University (Woakes et al., 1995). From these, 39 females returned to the island and 36 were caught at the colony 1 year later (see Guillemette and Pelletier, 2022 for details). Although implanted data loggers increased body mass by 1.2% in females, they were not associated with negative effects on the instrumented birds (see Guillemette et al., 2002 for an experimental test on female eiders; White et al., 2013 for a meta-analysis). Data loggers recorded *T*_b_ every 16 s. Only the data from the 12 loggers with a full year of recordings were used for this study (most loggers stopped recording after 7 months). This study constitutes a follow-up of Guillemette and Pelletier (2022) dealing with *T*_b_ and egg-laying of this species.

### Breeding phenology and migration

We established a study plot of about 0.5 ha containing about 80 nests on the island. Between 2003 and 2006, we visited the study plot daily from April 1st to May 31st, between 16h00 and 17h00, to determine laying dates and incubation periods of females. Our visits did not cause the incubating females to leave their nests unattended. Eiders generally leave unattended the first and second eggs (Bolduc and Guillemette, 2003), so the beginning of laying was defined as the day the first egg was laid. In some cases, the female was on the nest at the first visit. Hence, we used the observed hatching or departure date and then subtracted 30 or 31 days, respectively, to obtain the date of first egg laid (Guillemette, unpublished data). Alternatively, we put our hands below the attending female to count the number of eggs laid.

Spring migration phenology was based on the identification of the first migration flight achieved by each female based on the method of Pelletier et al. (2007) and Guillemette et al. (2016). In short, both the daily frequency of flight episodes and the average duration of flight episodes increase during migrations of female Common Eiders and is easily recognized on a plot of time spent flying in relation to calendar days (Guillemette et al., 2007). These migration events were associated with at least one flight of more than 30 min. We used the occurrence of such flights (30 min duration) as a cut-off point to differentiate migrating from non-migrating birds. Critical daylight was quantified separately for each female and was defined, based on a 3-day running average, as the date *T*_b_ started to rise monotonously from a seasonal low.

### Underlying assumptions

**FIGURE 1:**
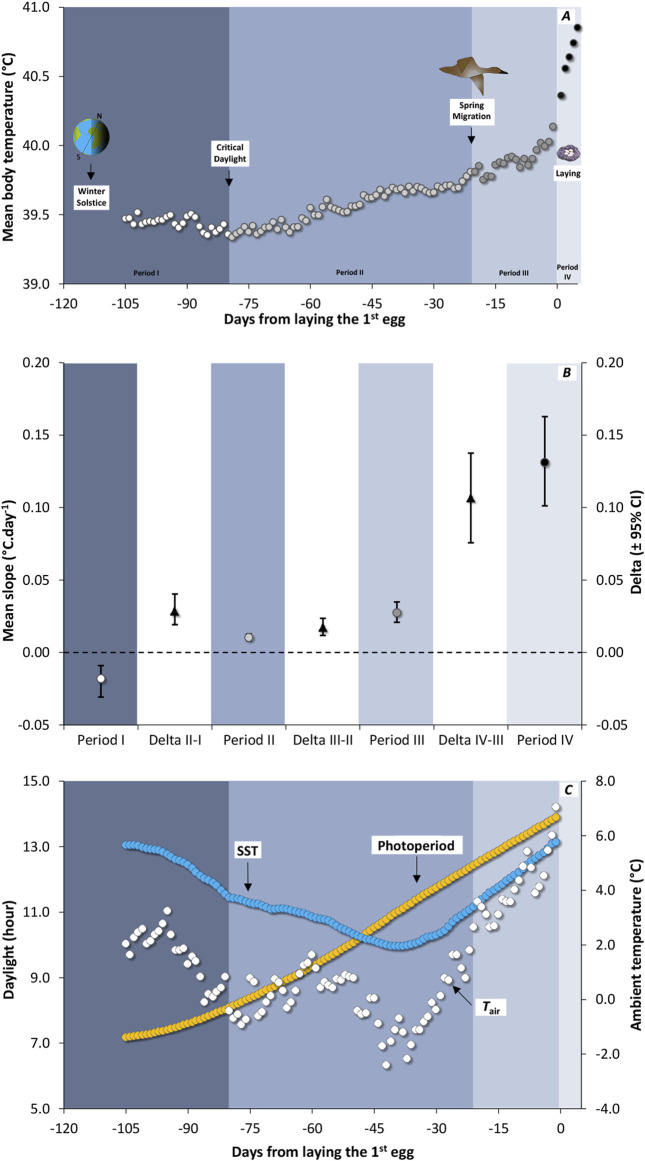
**(A)** Seasonal variation in daily core body temperature (*T*_b.daily_) from winter solstice to egg-laying in 12 female Common Eider *Somateria mollissima mollissima* instrumented in spring 2004 and 2005 and re-captured in spring 2005 and 2006. Each background color is associated with one of the four seasonal periods, with period I represented by white circles on a slate blue background, period II by light grey circles on a light steel blue background, period III by dark grey circles on a pastel lavender blue background, and period IV by black circles on a light sky grey background. *T*_b.daily_ was synchronised in relation to the laying of the first egg and averaged over the 12 females. Given the minimum time interval between winter solstice and laying date is 105 days (n < 12 females below that point), the average value is not shown over that point. **(B)** Average slope of *T*_b.daily_ variation over time (circles) for four different periods identified in **(A)** stemming from a reduced-major axis slope computed for each individual. The white band is showing the difference (average deltas with confidence intervals) between two adjacent seasons. When the bootstrap confidence intervals (95%) of the average difference between two periods (black triangles) exclude zero, the difference was declared significant. **(C)** Available daylight (photoperiod, yellow circles) and average variation in ambient temperature in relation to time. For both temperatures, sea surface temperature (SST; blue circles) and air temperature (*T*_air_; white circles) the average curves were computed from temperature in 2005 (5 females) and 2006 (7 females).

One underlying assumption of our approach is that daily core body temperature (*T*_b.daily_) does not vary in relation to locomotion activity performed by each individual. One such activity is flight, as it is associated with a large increase of power output and a rise of *T*_b_ (Guillemette et al., 2016). However, in their everyday life female eiders fly very little with only 1% of the time (Pelletier et al., 2008), without any impacts on *T*_b.daily_. This can be different for migration periods as flight time increase to 15% of the daily time budget (Guillemette et al., 2016). Although flight activity is causing large variation in *T*_b_, repeatedly within a migration day, it does not change *T*_b.daily_ between days. This is stemming from a Before-After analysis of *T*_b.daily_ showing that it changed, compared to the migration period, of only 0.05°C suggesting that migrating Common Eiders defend *T*_b.daily_ with success (Guillemette et al., 2017). Such a result is partly explained by the fact that flight-induced rise of *T*_b_ is closely regulated by cooling periods, on the water, between flights (Guillemette et al., 2017). Nevertheless, we tested this assumption by comparing the average *T*_b_ during the night, the inactive period, with average *T*_b_ during daylight (n = 12). Although *T*_b_ during daylight was 0.16°C higher than *T*_b_ during the night, both variables increase steadily (Guillemette, unpublished data) with photoperiod (r = 0.952 and r = 0.954, respectively) in a manner similar to Figure 1A.

### Data analysis

First, we averaged *T*_b_ for each day (*T*_b.daily_) and each female (5,400 datum per day) from winter solstice (December 21st) to the date of the last egg laid. Photoperiod (daylight = sunset - sunrise), *T*_b.daily_, and ambient temperature (SST and *T*_air_, see below) were associated by date for each female taken separately and then averaged over the 12 females. Then all females were synchronised in relation to the date of their first egg laid. Pearson correlations between *T*_b.daily_ and environmental variables (photoperiod, SST and *T*_air_) were computed as a first step on average values but were not tested for significance (due to potential autocorrelations in the time series, see Guillemette and Butler, 2012) followed by calculations of individual correlations (for each female taken separately). Using each individual correlation coefficient as a datum (see below), significance levels and confidence intervals (CIs) were conducted on average correlation coefficients (n = 12). For our location, we obtained SST data from the SST50 database of the NOAA (https://www.avl.class.noaa.gov/glossary/SST50.htm). The SST50 is defined as the daily mean SST in degrees Celsius at 1 m depth. Daily average *T*_air_ data were obtained from the nearby Rønne Airport located on Bornholm Island.

Seasonal *T*_b.daily_ decreases first from winter solstice to critical daylight and then increases in a non-linear fashion culminating during the laying period (Figure 1A). We thus calculated the slope of *T*_b_ variation, separately for each female, for four sections of the curve: (I) from winter solstice to critical daylight, (II) from critical daylight to spring migration, (III) from spring migration to the laying period, and then (IV) for laying period itself. The slopes of adjacent periods were then compared at the intra-individual level using a subtraction, giving one delta per individual. Deltas were averaged across the 12 females, for which 95% CIs were computed using the bootstrap method. We calculated CIs from 10,000 nonparametric bootstrap replicates using *Resampling Stats* software. When the CI of the average delta was excluding zero, the comparison was declared significant. Because some of the CIs resulting from the bootstrap method were slightly asymmetric, we reported minimum and maximum values for each average value.

Finally, lay dates of the 12 females were obtained over four consecutive years (2003–2006) for which we calculated the absolute time interval (days) between successive years. In a similar way, we calculated the relative time intervals between adjacent years, taking into account if the laying date of a particular female and year was later or earlier than the preceding year. In both cases, we computed the 95% CIs of the average value using the bootstrap method. Throughout the results section, results are presented with their 95% CIs in brackets.

## Results

In general, the mean *T*_b.daily_ of female eiders increased monotonously in relation to time (Figure 1A). Apart from a decrease of *T*_b.daily_ from solstice to an average low value occurring on January 19th (depicted as critical daylight), the rise in *T*_b_ accelerated over time (Figure 1B). For instance, the slopes of *T*_b_. daily were 0.010°C. day^-1^ (0.008, 0.013) for period II (from critical daylight to spring migration), 0.027°C. day^-1^ (0.021, 0.035) for period III (from spring migration to the beginning of laying), and 0.131°C. day^-1^ (0.101, 0.163) for period IV (during the laying period). As a result, the average *T*_b.daily_ increases by 1.5°C overall from critical daylight (39.4°C) to the end of laying period (40.9°C) over a period of about 90 days.

Using data only from the time of critical daylight, both photoperiod and mean *T*_b.daily_ increased in relation to time (Period II to Period IV, Figures 1A, C), resulting in a high average correlation coefficient (r = 0.971). When a similar relationship was calculated between mean *T*_b.daily_ and SST, a lower correlation coefficient (r = 0.383) was obtained. This is because SST is decreasing from critical daylight until the beginning of March while *T*_b.daily_ is increasing. For instance, SST started to increase about 40 days before the laying period. A higher correlation was obtained between *T*_b.daily_ and mean daily *T*_air_ (r = 0.700), the latter also reaching a seasonal low about 40 days before the laying period (Figure 1C), although it was more variable than SST.

**FIGURE 2:**
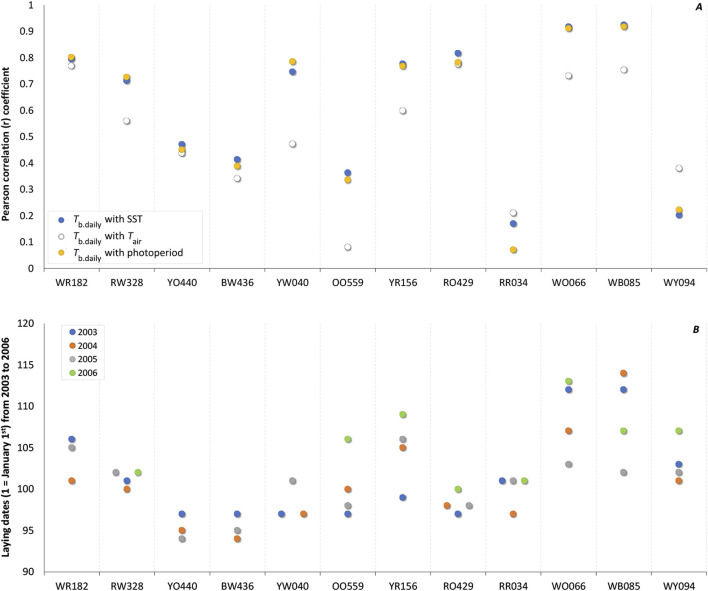
**(A)** Individual correlation coefficient (r) of daily body temperature (*T*_b.daily_) of Common Eider *Somateria mollissima mollissima* with sea surface temperature (SST; blue circles), air temperature (*T*_air_; white circles), and photoperiod (yellow circles) from 40 days before laying the first egg. When averaged over the 12 females together with 95% bootstrap confidence intervals (CIs), the correlation coefficients with *T*_b.daily_ are 0.609 (0.457, 0.750) for SST, 0.509 (0.381, 0.630) for *T*_air_, and 0.597 (0.435, 0.748) for photoperiod. **(B)** Laying date of the first egg of the 12 females over a period of 4 years (n = 44, 2003 in blue, 2004 in orange, 2005 in gray, and 2006 in green). The average absolute time interval (n = 32) from 1 year to the next was 3.22 days whereas the relative time interval was 0.66 days (see text).

Considering only these 40 days preceding the laying period, we obtained strong correlations with all environmental variables (SST and *T*_b.daily_: r = 0.940; *T*_air_ and *T*_b.daily_: r = 0.907; Photoperiod and *T*_b.daily_: r = 0.914). Although of a lower magnitude (Figure 2A), average individual correlations coefficients computed across the 12 females are significant for SST [r = 0.609 (0.457, 0.750)], *T*_air_ [r = 0.509 (0.381, 0.630)], and photoperiod [r = 0.597 (0.435, 0.748)]. This suggests the information potential of these three environmental variables as seasonal cues to be of similar level at this point in time (see Discussion).

We recorded the laying dates of these 12 females over 4 years (minus 4 missing records, n = 44) and the absolute time interval between 2 years for an individual female, was 3.22 days in average [(2.24, 4.20), n = 32] (Figure 2B). When taking into consideration if an actual date was earlier or later from a former date, we obtained a relative time interval and an average delta of -0.66 days [(−2.14, 0.83), n = 32].

## Discussions

Traditionally, time keeping mechanisms are studied from variation in reproductive organs (ovaries or testes; Bissonnette and Zujko, 1936; Farner, 1964; King et al., 1966; Tewary and Ravikumar, 1989; Silverin et al., 1993; Jain and Kumar, 1995; Huysentruyt et al., 2013). Here we present variation in *T*_b_ that parallel these observations indicating that *T*_b.daily_ may respond similarly to available daylight, and hypothesize that *T*_b.daily_ variation is endogenously programmed. Since we did not observe any influence of supplementary cues such as ambient temperature, we described the food system and quantified metabolic constraints of this species to support our discussion.

### *T*_b.daily_ and photoperiod: a potential mechanism

*T*_b.daily_ was highly correlated with photoperiod, the former starting to increase with time from the last third of January (Figures 1A, C). In the early 20th century, it was discovered that available daylight (i.e., photoperiod) triggers the timing of migration and breeding among wild bird populations (Rowan, 1925; Bissonnette, 1931). Experimental studies (Silverin et al., 1993; Trivedi et al., 2006; Dixit and Singh, 2011; Dawson et al., 2014; Eichhorn et al., 2021) confirmed these seasonal variations to be triggered by photoperiod. *T*_b.daily_ of female eiders was also accelerating at the approach of the breeding season and peaking during laying (Figure 1, see also Wascher et al., 2018). Such an observation is similar to the increases of reproductive organs of northern hemisphere birds (Farner, 1964; King et al., 1966; Tewary and Ravikumar, 1989; Silverin et al., 1993; Huysentruyt et al., 2013). For example, the slope of *T*_b.daily_ in relation to time is three times higher from spring migration to the start of the laying period (0.027°C. day^−1^) compared to the slow increase observed between the critical daylight and migration dates (0.010°C. day^−1^). Based on Guillemette and Pelletier (2022), we suggest that such an acceleration is associated with rapid follicular growth, which occurs about 10 days after arrival on the breeding grounds and lasts about 10 days for a clutch of five eggs in this species. The acceleration of *T*_b.daily_ is even more pronounced during the laying process with a slope of 0.131°C. day^−1^. In this latter case, the dramatic increase of *T*_b.daily_ is likely the outcome of secretion of progesterone (Bluhm et al., 1983; Sockman and Schwabl, 1999) and prostaglandin (Etches et al., 1990), two hormones associated with egg laying and thermogenic effects. Another mechanism that could drive such an acceleration of *T*_b.daily_ during egg-laying is the thermoregulatory adjustments associated with the increasing time spent in a terrestrial environment as laying progresses (see Guillemette and Pelletier, 2022).

Using thyroidectomy, Benoit (1935) discovered that thyroid hormones played a major role in triggering gonadal maturation of birds. Thereafter, the observation that thyroid hormones vary between reproductive and non-reproductive states of birds was confirmed several times from modern studies (Wieselthier and Van Tienhoven, 1972; John and George, 1978; Garbutt et al., 1979; Elarabany et al., 2012; Pérez et al., 2016; see reviews of Yoshimura, 2013; Dardente et al., 2014). Therefore, given the well-known thermogenic effect of triodothyronine (T3; McNabb, 2007) and the observed seasonal increase of plasma T3 preceding reproduction in some species (Jallageas et al., 1978; Jallageas and Assenmacher, 1979; Chastel et al., 2003), we hypothesize that *T*_b_ of wild birds might be altered in a seasonal fashion by thyroid hormones. The mechanism connecting thyroid hormones with seasonal reproduction of birds has been identified few years ago with the discovery that long days stimulate the synthesis of a specific enzyme (Type II iodothyronine deiodinase) into the medial basal hypothalamus of quails. This enzyme transforms the non-active thyroxine (T4) into its active form T3 (Yoshimura et al., 2003). The latter being the signalling hormone inducing the gonadotropin-releasing hormone cascade responsible for the onset of breeding. Such a mechanism has been confirmed in other avian species, including tits, sparrows and canaries (see Yoshimura, 2013 for a review).

### On the influence of supplementary cues

Although photoperiod is often considered as an initial predictive factor that initiates the neuroendocrine cascade for development of migration and breeding, supplementary factors (also referred to as local predictive factors) such as ambient temperature, and food abundance and quality may influence the more precise timing of the events (Ramenofsky, 2011). Given that *T*_b.daily_ is highly correlated with time, reaching an annual peak at egg-laying, it is appropriate to analyse the relationships between supplementary cues and *T*_b_ of females (Table 1).

**TABLE 1:** Potential importance of supplementary cues that may predict reproductive phenology of female Common Eiders *Somateria mollissima mollissima* breeding in the Baltic Sea.

Supplementary cue	Observations	Potential as a cue	References
Sea Surface Temperature (SST) from critical daylight to laying period	The correlation (r) between daily core body temperature (*T*_b.daily_) and SST is 0.383	Low	This paper
	The variation of this cue has a low incidence on the daily energy budget of eiders upon arrival at the colony	Low	Jenssen et al. (1989), This paper
Air temperature (*T*_air_) when on the water	The correlation between *T*_b.daily_ and *T*_air_ is 0.700	Medium	This paper
	Air is much less conductive than SST	Low	Jenssen et al. (1989), This paper
*T*_air_ when on the colony	Small probability of overheating on land while laying eggs	Low	Gabrielsen et al. (1991), Guillemette and Pelletier (2022)
Food abundance	In contrast to (more saline) oceanic environments, large biomass of Blue Mussels (*Mytilus edulis*) are available year-round, down to 40 m	Low	Wolowicz et al. (2006), Westerbom et al. (2019)
	Food type available to eider ducklings (<1 kg) is unknown. At one 1 kg of body mass, juvenile eiders feed on Blue Mussel	High to Low	Nyström et al. (1991)
Food quality	Maximum condition of Blue Mussels is reached 1 month before breeding (March) and lasts until June (spawning season) in the Baltic	Low	Wolowicz et al. (2006)

One would expect SST to be a major modulator of metabolic rate variation of aquatic birds. This is because water is highly conductive compared to air, together with the fact that legs and the beak (non-feathered organs) of aquatic species like the Common Eider, are regularly in contact with water. One main strategy endotherms may use when facing variation in ambient temperature is the regulation of heat exchange; thus they would minimise the difference between *T*_b.daily_ and SST (the Scholander-Irving model, see McNab, 2002). Therefore, it follows that cold SST should be matched by lower *T*_b.daily_. This is contrary to what was observed in this study (see Results). SST was decreasing until the beginning of March while *T*_b.daily_ started to increase from the last third of January, resulting in a negative relationship first (Figures 1A, C). Indeed, at about 40 days before laying the first egg, the relationship between SST and *T*_b.daily_ is reversing to a positive relationship. As a final note, it may be believed that female eiders in our study are idiosyncratic, as they do not appear to use ambient temperature, like SST, as a cue to initiate breeding. A recent meta-analysis of 61 marine bird species monitored between 1952 and 2015, during 18 years in average, found that SST did not influence the laying date of birds across a broad geographic area (Keogan et al., 2018).

Another argument for the role of SST on the phenology of breeding birds was proposed by Stevenson and Bryant (2000). They suggested that variation in ambient temperature (SST and *T*_air_) could influence the laying dates of birds as variation in temperatures would constrain the energy budget of birds. When calculating ambient temperature over a period of 10 years (2001–2011), the mean SST was 5°C at the average laying date (April 14th), ranging from 3.6°C to 7.4°C during that decade. Using the model of Jenssen et al. (1989), stemming from measurements of oxygen consumption of eiders at various temperatures on the water, it can be calculated for pre-laying females weighing 2.64 kg (Guillemette and Ouellet, 2005), that resting metabolic rate would be 1,147 kJ. day^−1^ and that 1°C difference in SST would represent a variation of about 20 kJ. day^−1^ (1.7% of resting metabolic rate). Using 1,028 kJ kg^−1^ as specific daily energy expenditure (Guillemette and Butler, 2012), we obtain that a variation of 1°C of SST would represent about 0.7% of the daily energy budget (2,714 kJ day^−1^). These calculations suggest altogether that variation in SST over the years is unlikely to have a substantial impact on the energy budget of breeding eiders and thus, would not have any impact on the phenology of breeding eiders.

Our data showed that it is unlikely that *T*_air_ is driving the seasonal variation of *T*_b.daily_ (but see Linek et al., 2021) given that *T*_air_ decreases while *T*_b.daily_ increases, until about 40 days before the laying period (Figure 1). This is in line with the low incidence of *T*_air_ compared to SST on the thermoregulation of marine birds while they float on the water (Jenssen et al., 1989). However, *T*_air_ could still play a role when females are reaching an annual maximum of *T*_b.daily_ while laying eggs at the colony (Guillemette and Pelletier, 2022). This could represent a thermoregulation challenge for these birds as any heat waves could jeopardize any breeding effort through overheating. This is unlikely for two reasons. First, because laying females are going back and forth between the water and the colony, Guillemette and Pelletier (2022) have tested the hypothesis that *T*_air_ is influencing the time of the day at which females are laying eggs. Using thermistors installed at the nest after the first egg was laid, they found that laying bouts at the colony occurred at a temperature of 6.5°C in average compared to when they were off their nest with a temperature of 4°C, which contradicts the above hypothesis. Second, two measures of lower critical temperature in air are available for this species, the first one being 0°C for winter-acclimatized birds (Jenssen et al., 1989) and the second one being 7°C for incubating females (Gabrielsen et al., 1991). Given that laying bouts at the colony also occur at about 7°C, this strongly suggest that the temperature range at which laying occurs in female eiders (at least at this colony) is safe in terms overheating probability.

However, it is often hypothesised that the main influence of ambient temperature on the phenology of laying dates is indirect through its impact on food abundance or quality (Shipley et al., 2020). Here we suggest that, at the time of breeding, it is unlikely that SST has any influence on Blue Mussel (*Mytilus edulis*) abundance (see Table 1), the main prey of pre-laying Common Eiders in the Baltic Sea (Öst and Kilpi, 1998). The two reasons are: (1) There is always a fairly large standing biomass remaining in the system despite the presumed impact of predation and physical factors (Westerbom et al., 2019). In fact, the Blue Mussel population of the Baltic Sea is different to other “oceanic” populations in the world as this area is characterised by a low salinity, which explains the occurrence of smaller mussels (Kautsky, 1982; Westerbom et al., 2002) and the absence of sea stars (and echinoderms in general), the main predator of oceanic Blue Mussels (Paine, 1974). Although the total biomass of mussels may vary from year to year (Westerbom et al., 2019), mussels are available along a depth gradient (down to 40 m; Wołowicz et al., 2006), which contrasts with truly marine environments where sea stars often limit mussels to shallow waters (Paine, 1974; Guillemette et al., 1996; Larsen and Guillemette, 2000). (2) Although there is no information about the diet of eiders ducklings (<1 kg) in the Baltic, which are guided by tending females on the feeding grounds after hatching (Bustnes and Erikstad, 1991), Nyström et al. (1991) have identified the main prey of juvenile eiders (at about 1 kg body mass) to be Blue Mussels in the Baltic. Nevertheless, eider ducklings form complex coalitions composed of multiple broods for which survival is typically low (about 10%–20% within the two first weeks) with about one good year for every decade (see review by Waltho and Coulson, 2015) and it has been suggested that body condition of breeding females is the main driver of ducklings’ survival, not food abundance (Bustnes and Erikstad, 1991; Kilpi et al., 2001).

Apart from the biomass of the main prey, one important feature for the energy intake of pre-laying Common Eiders is the energetic content of Blue Mussels (Guillemette, 2001). The condition of Blue Mussels varies dramatically in relation to spawning phenology in this species, which is apparently triggered by an early phytoplankton bloom occurring in the Baltic by the end of winter (Kautsky, 1982; Knöbel et al., 2021). As a result, flesh dry mass, lipid contents and gonadal index are at their peak for a period of 3 months (March-June) in the Baltic (Wołowicz et al., 2006), that is about 1 month before and 1 month after the breeding season has started in this colony of breeding eiders. In other words, the Baltic Sea seems to offer a perpetual and sufficient amount of biomass every year with a large window of maximum quality that encompasses the laying and incubation periods of this benthic predator. We thus conclude that inter-annual variations of SST in the Baltic Sea are unlikely to have any important influence on the food supply of Common Eiders at the time of breeding.

### Clockwork precision

One of the main arguments to discard the influence of photoperiod as a contributing cue at the moment of breeding is the observation that laying dates differ from year to year while photoperiod does not. Here we report the laying dates of the instrumented females monitored over 4 years where the variation ranged from 0 to 10 days with an absolute mean time interval among laying dates of 3.2 days and a mean adjacent difference between years of 0.66 days, with 7 females forming a tight cluster over the years (Figure 2B). Such variation in laying dates represent only a tiny variation in relation to a full year. This is, in our opinion, remarkable if we consider that most migrating birds needs to cope with various physiological and ecological challenges over the year. For example, (a) female eiders may spend a variable amount of time accumulating body reserves to recover from the incubation fast after breeding (Guillemette and Butler, 2012), (b) they may be delayed or hastened during migration by variable wind conditions (Day et al., 2004), they (c) may also postpone or advance the start of wing moult in relation to body mass (Guillemette and Butler, 2012; Viain and Guillemette, 2016), etc. We therefore speculate that the small variation observed in laying dates of our instrumented females might be the result of coping with or without success with these eco-physiological challenges occurring over the year.

In conclusion, based on the various mechanisms at work, we conclude that photoperiod was the main cue driving the seasonal breeding of eiders. We propose the observed seasonal increase of *T*_b.daily_ to operate through thyroid hormones, although the exact mechanism of action remains to be described. Finally, given that a photoperiod-driven breeding program would predict no or little variation in laying dates within individuals over the years, we suggest that the observed residual variation is the result of eco-physiological challenges occurring over the years.

## Data Availability

The raw data supporting the conclusions of this article will be made available by the authors, without undue reservation.
